# Maximizing utility of nondirected living liver donor grafts using machine learning

**DOI:** 10.3389/fimmu.2023.1194338

**Published:** 2023-06-29

**Authors:** Kiran Bambha, Nicole J. Kim, Mark Sturdevant, James D. Perkins, Catherine Kling, Ramasamy Bakthavatsalam, Patrick Healey, Andre Dick, Jorge D. Reyes, Scott W. Biggins

**Affiliations:** ^1^ Division of Gastroenterology and Hepatology, Department of Medicine, University of Washington, Seattle, WA, United States; ^2^ Center for Liver Investigation Fostering discovery (C-LIFE), University of Washington, Seattle, WA, United States; ^3^ Clinical and Bio-Analytics Transplant Laboratory (C-BATL), University of Washington, Seattle, WA, United States; ^4^ Division of Transplant Surgery, Department of Surgery, University of Washington, Seattle, WA, United States; ^5^ Pediatric Transplant Surgery Division, Department of Surgery, Seattle Children’s Hospital, Seattle, WA, United States

**Keywords:** anonymous, altruistic, living donation, allocation, liver transplant, allograft, artificial intelligence

## Abstract

**Objective:**

There is an unmet need for optimizing hepatic allograft allocation from nondirected living liver donors (ND-LLD).

**Materials and method:**

Using OPTN living donor liver transplant (LDLT) data (1/1/2000-12/31/2019), we identified 6328 LDLTs (4621 right, 644 left, 1063 left-lateral grafts). Random forest survival models were constructed to predict 10-year graft survival for each of the 3 graft types.

**Results:**

Donor-to-recipient body surface area ratio was an important predictor in all 3 models. Other predictors in all 3 models were: malignant diagnosis, medical location at LDLT (inpatient/ICU), and moderate ascites. Biliary atresia was important in left and left-lateral graft models. Re-transplant was important in right graft models. C-index for 10-year graft survival predictions for the 3 models were: 0.70 (left-lateral); 0.63 (left); 0.61 (right). Similar C-indices were found for 1-, 3-, and 5-year graft survivals. Comparison of model predictions to actual 10-year graft survivals demonstrated that the predicted upper quartile survival group in each model had significantly better actual 10-year graft survival compared to the lower quartiles (p<0.005).

**Conclusion:**

When applied in clinical context, our models assist with the identification and stratification of potential recipients for hepatic grafts from ND-LLD based on predicted graft survivals, while accounting for complex donor-recipient interactions. These analyses highlight the unmet need for granular data collection and machine learning modeling to identify potential recipients who have the best predicted transplant outcomes with ND-LLD grafts.

## Introduction

**Figure 1:**
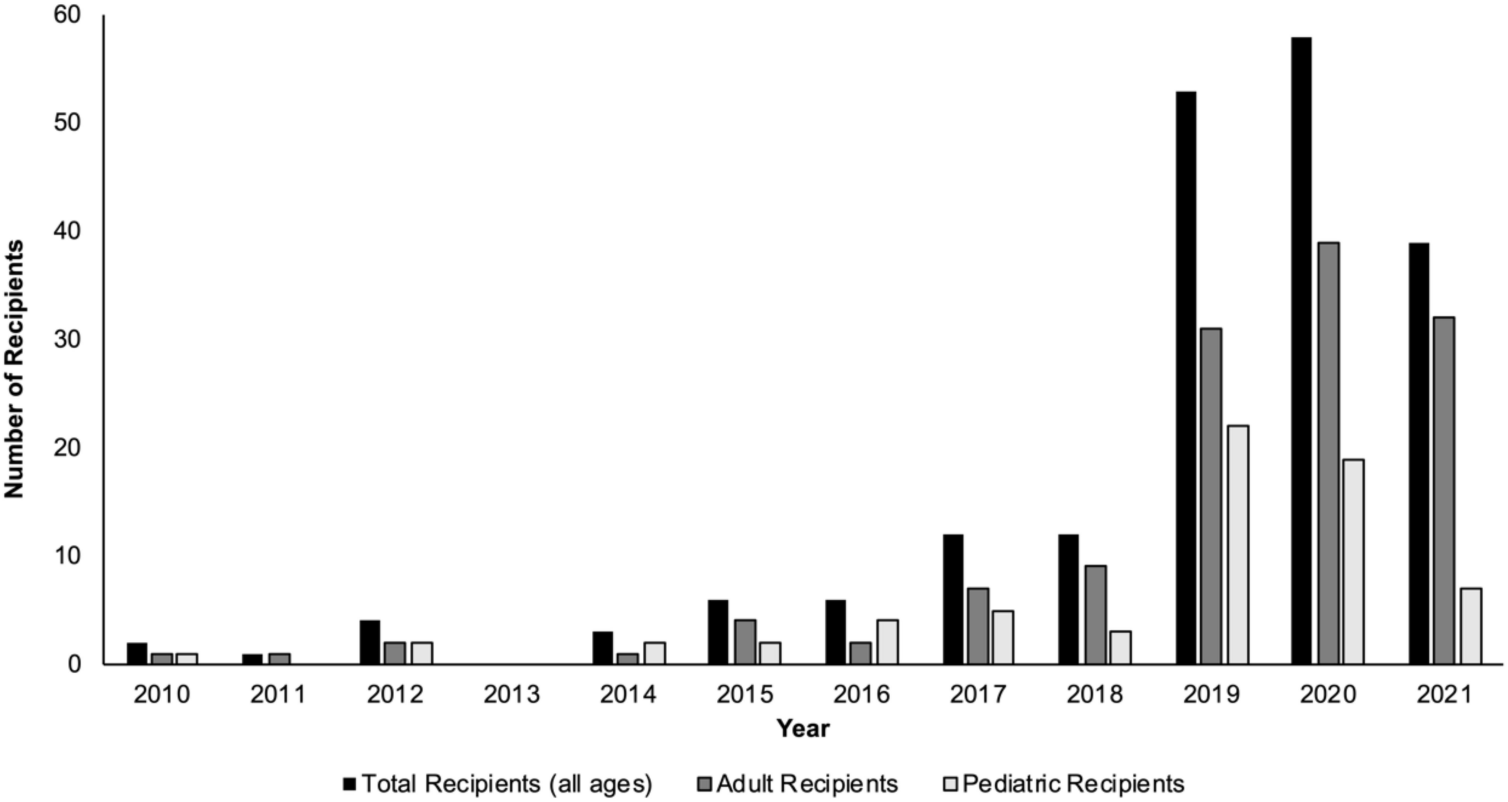
Nondirected living liver donor transplant in the U.S. (2010-2021).

In the past decade, the number of living donor liver transplants (LDLTs) in the United States, along with the number of transplant centers performing LDLTs, have both increased (1–3). Over the course of 2010-2018, the mean annual number of LDLTs performed was 264; however, from 2019-2020, that mean annual number increased to 507 (1). Although the majority of living liver donors in the U.S. are ‘directed’ living donors (i.e., the living liver donor and intended recipient have a known affiliation or relationship pre-transplant), the number of nondirected living liver donors (ND-LLD, living liver donors who are willing to donate anonymously to anyone in need) has been steadily increasing in the U.S. over the past several years (**Figure 1**) (1, 4–6).

Living donors interested in nondirected liver donation frequently express desire for their donation to affect the greatest good from the graft. ND-LLD grafts offer transplant centers the opportunity to help patients who may be underserved by the current deceased donor Model for End-Stage Liver Disease (MELD)-based allocation system, for example, individuals with small stature or low MELD patients with portal hypertensive complications. Additionally, ND-LLDs enable liver transplant centers to consider more novel undertakings such as living liver donor chains. Despite the recognized importance of ND-LLDs, there is no standardized evidence-based approach to help guide liver transplant centers in ND-LLD graft allocation. Currently, individual centers develop their own internal preferences and policies regarding how to internally allocate liver grafts from ND-LLDs, with all U.S. programs abiding by the the National Organ Transplant Act that was implemented in 1984, making it illegal to buy or sell human organs and tissues in the U.S. United Network for Organ Sharing (UNOS) policy 14.6.B allows for utility-based allocation processes, and guidance from the Organ Procurement and Transplantation Network (OPTN) recommends that liver transplant centers ‘make an effort to match donors and candidates appropriately’ and ‘maximize the potential good’ that will emerge from ND-LLD grafts (7). We therefore aimed to develop analytic models predicting graft survival to help inform ND-LLD graft allocation.

## Materials and methods

### Data source and variables

We conducted a retrospective analysis of the OPTN liver dataset. The dataset contained information on U.S. recipients who underwent liver transplant from living liver donor grafts from 1/1/2000 to 12/31/2019, with follow-up to 3/20/2020. Data from both directed and nondirected living liver donation was collected. Donor and recipient data were recorded from the transplant candidate and transplant recipient forms. We excluded recipients of deceased donor grafts, whole liver grafts, and multiorgan transplants.

The donor data collected included: age at donation, sex, race, history of cigarette smoking, ABO blood type, height (cm), weight (kg), and calculated donor body surface area [BSA, m (2)] using the formula by Mosteller (8). Although graft-to-recipient weight ratio (GRWR) is not included in the OPTN dataset, BSA was included in our analyses since prior studies have demonstrated an increased risk of graft failure when donor and recipient BSA are mismatched (9, 10). Transplant factors recorded included: type of hepatic graft (right [segments 5-8], left [segments 2-4], and left-lateral [segments 2 and 3]) and cold ischemia time in hours (CIT, length of time from when the donor organ is flushed with cold solution until it is removed from ice just prior to anastomosis in the recipient). The majority of living liver donor grafts (75%) in the OPTN data had CIT > 1 hour. We used a cut-off of 6-hours based upon our statistical analyses demonstrating that living liver donor grafts with >6 hours of CIT had a worse survival compared to all the other CIT cut-offs (for example, 0-1 hours, 1-2 hours, 2-3 hours, etc).

Recorded recipient factors included: age at time of transplant (adults defined as >/=18 years; children defined as <18 years), sex, height (cm), weight (kg), calculation of recipient BSA, etiology of underlying liver disease, calculated MELD score or Pediatric End-Stage Liver Disease (PELD) score at transplant, serum albumin level at transplant, presence of ascites (absent, slight, or moderate, as recorded in the OPTN database), encephalopathy (none, grade 1-2, or grade 3-4, as recorded in the OPTN database), presence of portal vein thrombosis, previous abdominal surgery, transjugular intrahepatic portosystemic shunt, diabetes mellitus diagnosis, location at time of transplant (intensive care unit [ICU], inpatient [non-ICU], outpatient), need for mechanical ventilation and/or pressor support, on dialysis the week prior to transplant, Epstein-Barr virus serostatus, and Cytomegalovirus serostatus.

The dataset used to develop the analytic models described in this study is available through the OPTN.

All data obtained from human participants were obtained in accordance with the ethical standards of the institutional and/or national research committee and with the 1964 Helsinki Declaration and its later amendments or comparable ethical standards.

The interpretation and reporting of these OPTN data are the responsibility of the authors and in no way should be considered an official policy of, or interpretation by, the OPTN or the U.S. Government. The University of Washington Human Subjects Division deems that the OPTN database is de-identified and publicly available, and thus this study was exempt from human subjects review.

### Statistical analyses

Continuous variables were depicted as median and interquartile ranges (IQR). Categorical variables were presented as percentages. For all data, if <1% of the categorical values were missing, the majority value was given. For the 305 donors and 66 recipients with missing body weight, values were imputed with linear regression using age, sex, and race. For the 66 donors and 16 recipients with missing height, values were imputed with linear regression using age, sex, and race. For the 111 recipients missing albumin level, the median albumin value of 3.1 was given. For 107 recipients with missing values for ascites, the value of ‘absent’ was given. Sensitivity analyses revealed no change in the final results by imputing any of the values. Chi-square analysis was used to compare categorical variables. One-way ANOVA was used to compare continuous variables. Donor-to-recipient BSA ratio (D-R-BSA) was calculated for each donor-recipient pair. Determining the critical ratio (or cut-off) for D-R-BSA for each graft was performed using multiple methods (11, 12). Multiple ranges were compared with Cox proportional hazard models and many chi-square analyses to determine the best critical ratio for D-R-BSA with significant graft loss. Through these methods, we determined the critical ratios for D-R-BSA, with increasing graft loss occurring below 2.0, 1.5, and 1.0 for the left-lateral, left, and right grafts, respectively.

### Model development

Machine learning survival models are increasingly being utilized in the field of transplantation (13). These models are not restricted by the rules of the Cox proportional hazard model, like the assumption that variables must have hazards functions that are proportional over time, or the relationship between the log hazard and each variable being linear, or collinearity between variables, or finding numerous interactions. We used the machine learning survival model of Random Forest Survival (RFS). It is an ensemble model (averaging of many models) of tree-based learners. Tree-based learners, also known as decision trees, develop many splits or decision rules of the data to determine which characteristics influence survival. After performing many trees, the ensemble method averages all the trees to determine the best splitting rules for the data. A formula is not created with an RFS model, but rather variables of importance can be given for each model. The importance measure of a variable is calculated by how much the accuracy decreases when the variable is excluded in reference to other variables in the model (14, 15). The specific value of the variable importance is not intrinsically meaningful and depends on the number of variables in the algorithm and the number of trees evaluated in the model. Therefore, the value of the variable is interpreted relative to the values of other variables. For example, a variable with an importance value of 0.20 is two times more predictive than a variable with the value of 0.10. Despite increasing use of machine learning in the medical field, the actual machine learning models may be perceived by readers to be ‘black boxes’ that appear to be difficult to interpret unless the model is explained and interpreted correctly. Therefore, to explain the model and to increase model transparency and interpretability of the results, we graphed LIME (Local Interpretable Model-agnostic Explanation) plots, where the output of LIME is a list that reflects the contribution of each variable to the prediction of a data sample. In our LIME plots, values for variable importance that are graphed as negative numbers connote a decrease in graft failure risk, whereas values for variable importance that are graphed as positive numbers connote increased graft failure risk (16). In this model, when comparing negative and positive important values, the absolute values are to be used.

In our analyses, RFS models were used to predict 10-year graft survival, and variable selection was constructed to optimize the concordance index (c-index), which is the proportion of observations that the statistical model can order correctly in terms of survival times and, for binary outcomes, may be considered conceptually similar to the area under the receiver operating characteristic curve (AUC) (17, 18). Due to the marked difference in characteristics between the three graft types, a separate RFS model predicting 10-year graft survival was constructed for each graft type. Importantly, it is not the intention of the model to determine which hepatic lobe should be donated, since that is a transplant surgical clinical decision that is based upon the surgeon’s assessment of the donor’s vascular and biliary anatomies, hepatic volumes, and discussions with the donor. Rather, the intention of the model is to help liver transplant programs identify potential recipients for specific grafts, based on maximizing graft utility, for the non-directed allograft donation. Data for each graft type was split (70%/30%) into a training set and test set, respectively, while maintaining the same percentage of graft failures in each proportion. Survival for predicted groups were calculated by Kaplan-Meier survival analysis and compared by log-rank test. All results were considered significant with a *p*-value <0.05. We also modeled 5-year, 3-year- and 1-year graft survivals, and found similar c-indices as for 10-year graft survival (data not shown). The Chi-square, ANOVA, Kaplan-Meier, and Cox proportional hazard analyses were performed using JMP-Pro Version 15.1.0 (SAS Institute, Inc., Cary, NC, USA). The RFS analyses were performed using R version 4.0.0. and the randomForestSRC and the random package (19).

## Results

### Descriptive statistics by graft type

We included data on all 6328 living donor liver transplant recipients from 1/1/2000 to 12/31/2019. LDLT from non-directed donors accounted for 5% of total LDLDs during the study period. There were 1063 left-lateral, 644 left, and 4621 right grafts. Of the left-lateral grafts, 1001 (94%) were transplanted into recipients aged 0-12 years, and 62 (6%) transplanted into older recipients (> 12 years). Of the left grafts, 213 (33.0%) were transplanted into recipients aged 0-17 years, while 431 (67%) were transplanted into older recipients (>17 years). Of the right grafts, 100 (2%) were transplanted into recipients aged 0-17 years, compared to 4521 (98%) transplanted into older recipients (>17 years). More females received left grafts (61%), whereas more males (58%) received right grafts. Patients with cholestatic liver diseases (primary biliary cirrhosis [PBC] and primary sclerosing cholangitis [PSC]) accounted for 24% of left and 24% of right graft recipients. The D-R-BSA ratio was significantly different (*p*<0.001) between all graft types, with D-R-BSA ratios (median and IQR) of 4.3 (3.1-5.3), 1.21 (1.1-1.5), and 1.0 (0.9-1.1) for the left-lateral, left, and right grafts, respectively. Other donor and recipient characteristic comparisons by graft type (left-lateral, left, and right) are provided in **Table 1**. Due to the large difference in recipients’ ages at transplant, and given that the majority of left lateral grafts are transplanted into young, pediatric recipients and the majority of right grafts are transplanted into adult recipients, most of the other recipient characteristics were significantly different between graft types.

**Table 1:** Donor and recipient characteristics of living donor liver transplants performed 1/1/2000-12/31/2019.

	Type of Hepatic Graft			
Characteristic	Left-Lateral (N=1063)	Left (N=644)	Right (N=4621)	P Value
Donor				
Age, years (median, IQR)	32(26-37)	34.5(27-43)	36(29-45)	<0.001
Donor Age Groups, years				
18-35	741(69.7%)	340(52.8%)	2160(46.7%)	<0.001
36-50	302(28.4%)	240(37.3%)	1939(42.0%)	<0.001
51+	20(1.9%)	64(9.9%)	522(11.3%)	<0.001
Race				
Asian	60(5.6%)	38(5.9%)	103(2.2%)	<0.001
Black	100(9.4%)	23(3.6%)	176(3.8%)	<0.001
Hispanic	188(17.7%)	86(13.4%)	514(11.1%)	<0.001
Other	17(1.6%)	5(0.8%)	64(1.4%)	0.30
White	698(65.7%)	492(76.4%)	3764(81.5%)	<0.001
Female Sex	617(58.0%)	285(44.3%)	2425(52.5%)	<0.001
Cigarette History				
No	609(57.3%)	398(61.8%)	2331(50.4%)	<0.001
Unknown	295(27.8%)	116(18.0%)	805(17.4%)	<0.001
Yes	159(15.0%)	130(20.2%)	1485(32.1%)	0.02
Cold Ischemia Time Groups, hours				
0-6	873(82.1%)	533(82.8%)	3681(79.7%)	0.06
6+	30(2.8%)	20(3.1%)	105(2.7%)	0.27
Unknown	160(15.1%)	91(14.1%)	835(18.1%)	0.005
Recipient				
Age, years (median, IQR)	1(0-2)	42(13-58)	53(45-60)	<0.001
Recipient Age Groups, years				
0-12	1001(94.2%)	160(24.8%)	54(1.2%)	<0.001
13-17	14(1.3%)	53(8.2%)	46(1.0%)	<0.001
18-40	8(0.7%)	98(15.2%)	777(16.8%)	<0.001
41-55	20(1.9%)	138(21.4%)	1770(38.3%)	<0.001
56+	20(1.9%)	195(30.3%)	1974(42.7%)	<0.001
Female Sex	545(51.3%)	393(61.0%)	1941(42.0%)	<0.001
Diagnosis				
AHF	112(10.5%)	39(6.1%)	112(2.4%)	<0.001
AIH	8(0.8%)	26(4.0%)	170(3.7%)	<0.001
Biliary Atresia	591(55.6%)	94(14.6%)	41(0.9%)	<0.001
Benign Tumor	5(0.5%)	3(0.5%)	27(0.6%)	0.85
Cancer (HCC and others)	65(6.1%)	87(13.5%)	627(13.6%)	<0.001
Cholestasis (PBC/PSC)	73(6.9%)	152(23.6%)	1095(23.7%)	<0.001
Alcohol	5(0.5%)	40(6.2%)	468(10.1%)	<0.001
Metabolic	78(7.3%)	27(4.2%)	123(2.7%)	<0.001
NASH/Cryptogenic	15(1.4%)	53(8.2%)	689(14.9%)	<0.001
Other	56(5.3%)	42(6.5%)	102(2.2%)	<0.001
Re-transplantation	39(3.7%)	9(1.4%)	57(1.2%)	<0.001
Viral	16(1.5%)	72(11.2%)	1110(24.0%)	<0.001
Any Type of Diabetes Mellitus	46(4.3%)	97(15.1%)	1008(21.8%)	<0.001
Medical Condition				
Home	585(55.0%)	504(78.3%)	4009(86.8%)	<0.001
In Hospital	253(23.8%)	90(14.0%)	490(10.6%)	<0.001
ICU	225(21.2%)	50(7.8%)	122(2.6%)	<0.001
Mechanical Ventilation and/or Pressor Support	117(11.0%)	28(4.4%)	67(1.5%)	<0.001
PELD/MELD Groups				
11 to 14	374(35.2%)	301(46.7%)	1832(39.6%)	<0.001
15 to 22	214(20.1%)	191(29.7%)	1579(34.2%)	<0.001
23 to 30	161(15.2%)	65(10.1%)	395(8.5%)	<0.001
31 to 40	314(29.5%)	87(13.5%)	815(17.6%)	<0.001
Serum Albumin Level	3.09 ± 0.75	3.18 ± 0.71	3.1(2.6-3.5)	<0.001
Ascites				
Absent	692(61.1%)	345(53.6%)	1552(33.6%)	<0.001
Slight	178(16.8%)	193(30.0%)	2025(43.8%)	<0.001
Moderate	193(18.2%)	106(16.5%)	1044(22.6%)	<0.001
Dialysis Week Before Transplant	14(1.3%)	10(1.6%)	35(0.8%)	0.07
Encephalopathy Grade				
None	715(67.3%)	388(60.3%)	1811(39.2%)	<0.001
1-2	94(8.8%)	179(27.8%)	1978(42.8%)	<0.001
3-4	43(4.1%)	19(3.0%)	175(3.9%)	0.47
Unknown	211(19.9%)	58(9.0%)	657(14.2%)	<0.001
Portal Vein Thrombosis	44(4.1%)	48(7.5%)	331(7.2%)	<0.001
Previous Abdominal Surgery	617(58.0%)	336(52.2%)	2000(43.3%)	<0.001
TIPS	11(1.0%)	49(7.6%)	402(8.7%)	<0.001
EBV Positive	287(27.0%)	321(49.8%)	3052(66.0%)	<0.001
CMV Positive	313(29.4%)	288(44.7%)	2430(52.6%)	<0.001
Combination Donor to Recipient				
Donor to Recipient BSA Ratio (median, IQR)	4.3(3.1 - 5.3)	1.21(1.1-1.5)	1(0.9 - 1.1)	<0.001
Critical Ratio (CR) for Donor to Recipient BSA	CR <2 102(9.6%)	CR < 1.5 475(73.8%)	CR < 1 2401(52.0%)	<0.001
ABO Match				
Identical	826(77.7%)	518(80.4%)	3624(78.4%)	0.39
Compatible	216(20.3%)	123(19.1%)	962(20.8%)	0.52
Incompatible	21(2.0%)	3(0.5%)	35(0.7%)	0.002
Graft Loss	194(18.3%)	188(29.2%)	1308(28.3%)	<0.001

AHF, acute hepatic failure; AIH, autoimmune hepatitis; BSA, body surface area; CMV, cytomegalovirus; CR, critical ratio for donor-to-recipient body surface area ratio; EBV, Epstein Barr virus; HCC, hepatocellular carcinoma; ICU, intensive care unit; IQR, interquartile range; MELD, Model for End-Stage Liver Disease score; NASH, nonalcoholic steatohepatitis; PBC, primary biliary cholangitis; PELD, Pediatric End-Stage Liver Disease score; PSC, primary sclerosing cholangitis; TIPS, transjugular intrahepatic portosystemic shunt.

### Kaplan-Meier graft survivals

**Figure 2:**
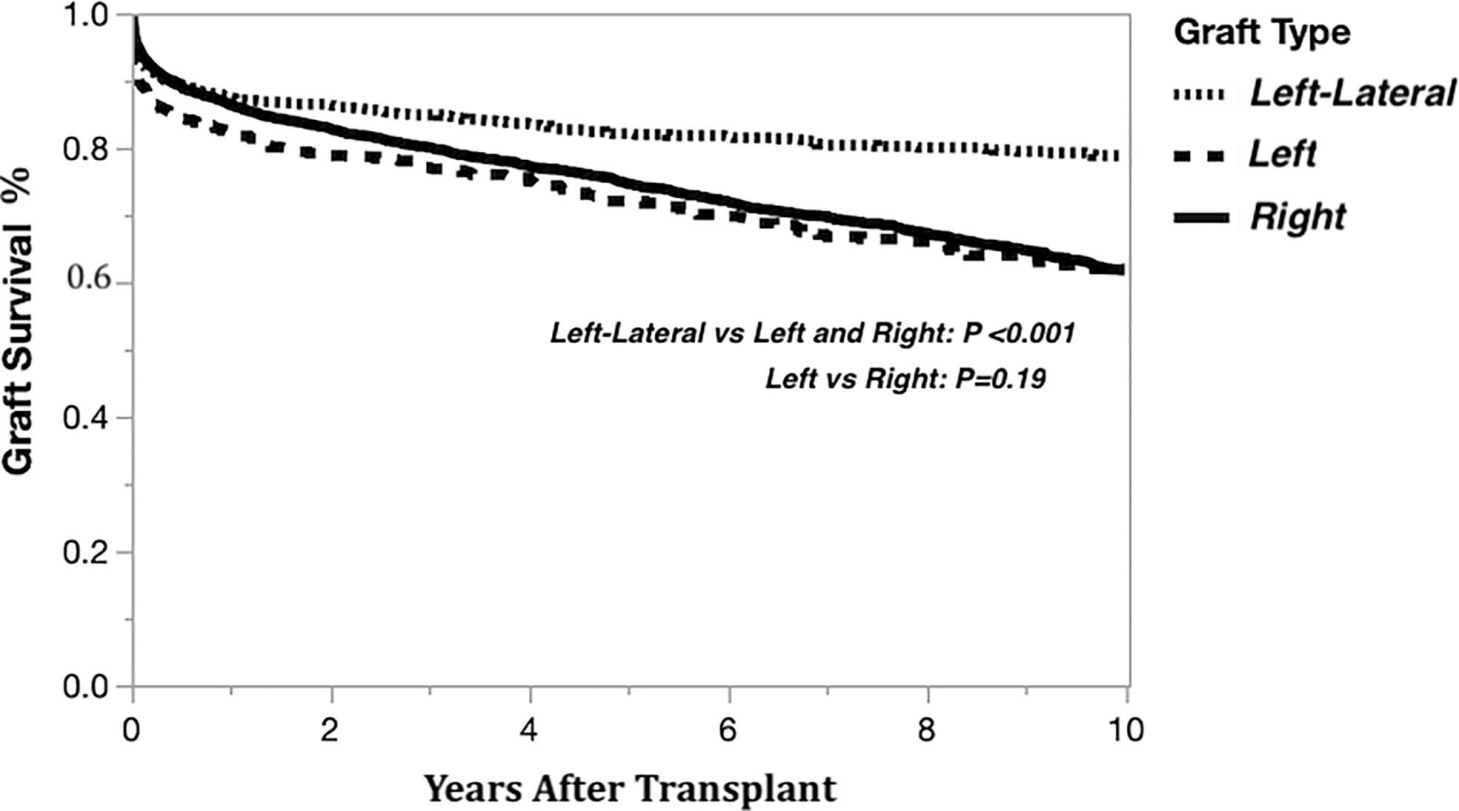
Kaplan-Meier actual graft survival curves derived from living donor liver transplants performed 1/1/2000-12/31/2019 by graft type.

**Figure 3:**
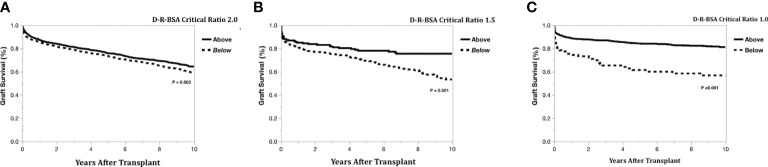
Kaplan-Meier actual graft survival curves confirming critical donor-to-recipient body surface area ratio. **(A)** Left-lateral graft, **(B)** Left graft, **(C)** Right graft.

The Kaplan-Meier graft survival curves comparing the three graft types are shown in **Figure 2**. The 10-year post transplant graft loss was significantly different (*p*<0.001) between graft types, with 194 (18%) losses for the left-lateral graft, compared to 188 (29%) and 1308 (28%) for the left and right grafts, respectively. There was no difference in graft survival when comparing left to right grafts. The Kaplan-Meier graft survival curves confirming the importance of the critical ratio for D-R-BSA (a surrogate for GRWR, which is not available in the OPTN data) for each graft type are shown in **Figure 3A** (left-lateral, critical ratio 2), **Figure 3B** (left, critical ratio 1.5), and **Figure 3C** (right, critical ratio 1.0). Graft survivals were lower when the D-R-BSA was below the critical ratio. All survival curves were significantly different when comparing survival above and below the critical ratio for D-R-BSA (*p*-value 0.002 for left-lateral, *p*=0.001 for left, *p*>0.001 for right).

### Random forest survival models by graft type

**Figure 4:**
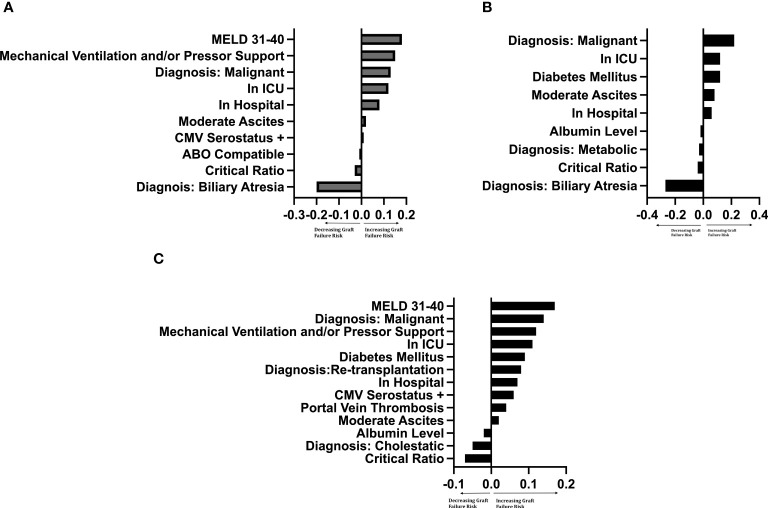
LIME plot of variables included in each graft survival prediction model after controlling for significant^†^ donor variables by importance ^‡†^Controlled for donor age (years), race, sex, cigarette history, cold ischemia time, CMV status. Critical Ratio: refers to critical level of donor-to-recipient body surface area ratio. ICU, intensive care unit. MELD, Model for End-Stage Liver Disease score. ^‡^The importance of a variable was determined by leaving the variable out of the model and evaluating how this removal influenced the accuracy of the model compared to other variables. The intrinsic value has no specific meaning, but the ratio of values between variables determines the relative importance of that variable to other variables. **(A)** Left-lateral graft, **(B)** Left graft, **(C)** Right graft.

The importance of the selected recipient and donor-to-recipient variables using random forest survival models is demonstrated graphically by LIME plots in **Figure 4**. As mentioned previously, the importance measure is calculated by how much the accuracy of the model decreases when the variable is excluded in reference to other variables in the model. The specific value of the variable is not intrinsically meaningful, but rather is interpreted relative to the values of other variables. The critical ratio for D-R-BSA was important for prediction in all three graft models, with critical ratios above the defined thresholds (i.e., ≥2.0 for left-lateral graft, ≥1.5 for left graft, and ≥1.0 for right graft) being of relatively high importance in the right graft model, and of lesser importance in the left and left-lateral graft models, relative to the other variables in each model. The other variables important in predicting 10-year graft survival in all three graft models were: malignant diagnosis, location at time of transplant (inpatient [non-ICU] or ICU), and having moderate ascites. The diagnosis of biliary atresia was of high importance in the left-lateral and left graft models, but was of no importance and not included in the right graft model. A diagnosis of cholestatic liver disease (PBC or PSC) was important in the right graft model, but not in the left-lateral and left graft models. The recipient being a re-transplantation candidate was only important in the right graft model. The best c-index was 0.70 for the left lateral graft model. The left and right graft models had c-indices of 0.63 and 0.61, respectively. Modeling of 5-year, 3-year- and 1-year graft survivals revealed similar c-indices as for 10-year graft survival (data not shown).

**Figure 5:**
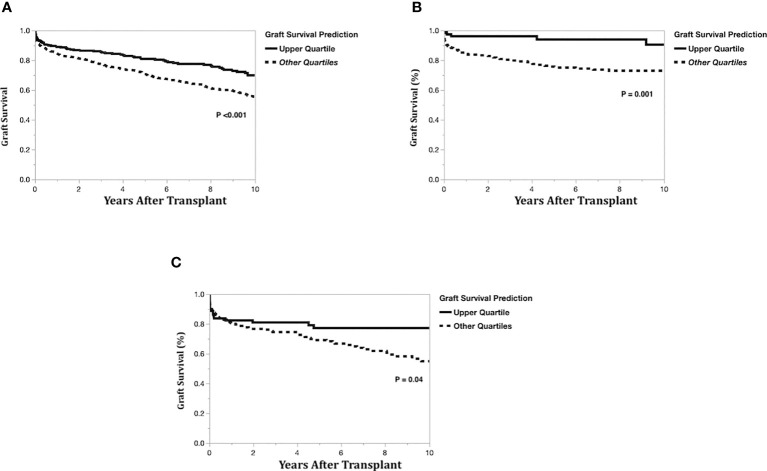
Kaplan-Meier actual graft survival curves by graft survival prediction quartiles. **(A)** Left-lateral graft, **(B)** Left graft, **(C)** Right graft.

To compare model predictions to actual graft survival, the models’ 10-year graft survival predictions were divided into groups consisting of the upper quartile and the lower three quartiles. The Kaplan-Meier actual graft survival curves for each model prediction group (upper quartile vs lower quartiles) are shown in **Figure 5A** (left-lateral graft), **Figure 5B** (left graft), and **Figure 5C** (right graft). For the left lateral, left and right graft modeling scenarios, the upper quartile prediction group had statistically significantly better 10-year graft survival than the lower three quartiles group (*p*<0.05), indicating that the model predictions have good discrimination for identifying which recipients will maximize long-term graft survival.

### Real-world implementation of our utility-based nondirected living liver donor graft allocation model

At the University of Washington, we began using this utility-based model in 2020 to assist our team in making evidence-based, less subjective decisions regarding allocation of hepatic allografts from nondirected living liver donors. Our process is not intended to be based solely upon the modeling results, making the actual values of the modeling c-indices less intrinsic to the selection process. Our approach to non-directed donor allocation incorporates our objective modeling results into our teams’ clinical assessments of potential recipients. Therefore, our decision-making process proceeds as follows: (1) using our liver transplant program’s list of patients who are active on the UNOS waiting list, we run our utility-based allocation model for the nondirected donor, taking into the lobe of liver being donated (left lateral, left or right lobe) and accounting for blood type compatibility between the nondirected donor and potential recipients. Additionally, our transplant surgeon specifies body size parameters for potential recipients (i.e., maximum body weight) based upon the donor’s graft volume; (2) the list of potential recipients generated from step 1 is rank ordered (descending) according to predicted probability of 10-year graft survival, with 65% as our lower limit cutoff for 10-year graft survival; (3) separately, a match run using our nondirected donor is conducted; (4) the rank ordered list generated from our utility model in step 2 is reviewed in a thoughtful multi-disciplinary clinical forum attended by our transplant hepatologists and transplant surgeons to review each individual potential recipient’s clinical indications for liver transplant, portal hypertensive complications, and surgical complexity that might impact suitability for receiving a living donor hepatic allograft. When there is more than one suitable potential recipient for the nondirected living liver donor graft, then the match run sequence is used to prioritize the potential recipients (a lower match sequence number confers higher priority).

To further characterize the potential recipients identified by our utility models, we compared these individuals to the potential recipients identified on the match run list. In this comparison, we found that potential recipients identified via the utility model had lower match MELD scores (16 [IQR 13-20] vs 21 [IQR 17-25]; p<0.001), lower BMIs (27.5 [IQR 24.1-32.5] vs 29 [IQR 25-34]; p=0.02), and similar Donor-to-Recipient BSA ratios (1.9 [IQR 1.7-2.1] vs 2.0 [IQR 1.8-2.3]; p=0.049). There were no significant differences in recipient characteristics between the utility model and match run with regards to: age, height, weight, sex, cholestatic liver disease diagnosis, or diagnosis of HCC (data not shown). Our nondirected donors have all been right-lobe donors thus far, but a similar process to that outlined above could be implemented for left or left lateral segment donors.

## Discussion

Nondirected (also known as ‘anonymous’) living liver donors present an important opportunity for expanding the donor organ pool. Currently, there is an array of terminology used in the living liver donor literature to describe these donors; therefore, to avoid confusion in this study, we have coined the term nondirected living liver donor (ND-LLD) to clearly represent the donor population to which we are referring. We present an original, proof of concept, evidence-based model to help identify and stratify potential recipients of ND-LLD livers, based on predicted 10-year graft survival, while accounting for complex donor-recipient interactions. We propose that this model is best applied to help guide thoughtful clinical decision-making, rather than advocating that any modeling results be used in isolation. Our model best predicted 10-year graft survival for left-lateral grafts (c-index 0.7) followed by left grafts (c-index 0.63) and right grafts (c-index 0.61). Model predictions correlated with actual recipient survival, indicating good discrimination for predicting which recipients will maximize liver graft survival. Our models are useful for objectively guiding ND-LLD graft allocation in the clinical setting. Our clinical liver transplant team has found this allocation process to be very efficient and beneficial in ensuring that we are evidence-based in the selection of a potential recipient, aiming to maximize the utility of the generous gift of the living liver donor’s graft. Additionally, our objective allocation process allows us to be transparent with our non-directed living liver donors when we discuss how the graft will be allocated, fostering increased trust between our transplant program and the donor. While we chose our desired outcome measure to be 10-year graft survival in an effort to maximize utility of the graft, we also investigated 5-, 3- and 1-year graft survivals and found similar modeling performances as in our 10-year outcomes models, so only our 10-year data are presented in detail in this manuscript.

To our knowledge, this is the first model using machine learning techniques to incorporate objective donor, recipient, and combined donor-to-recipient characteristics available in the OPTN data to predict long-term graft survival after LDLT. We present the results from our RFS models, though we did evaluate several other machine-based learning models (data not shown). The RFS models were the best models for the UNOS dataset and the models with the best validation.

Importantly, our models incorporate donor-to-recipient BSA ratios, which estimate liver volume more accurately than donor and recipient body weight and height alone (9, 10, 20). Our models also predict long-term 10-year graft survival for three different types of grafts. Although a number of scoring systems such as the MELD score (21), donor age-MELD (D-MELD) score (22), balance of risk score (BAR) (23), and the transplant risk index (TRI) (24), have been found to reasonably predict survival post-LDLT with AUC values of 0.60-0.70, these scoring systems have been limited to predicting short-term (up to 1-year) survival (25). Goldberg et al. also described the Living Donor Risk Index (LDRI), but this score had AUC values of 0.59-0.62 and only predicted graft survival up to 5-years post-LDLT (26). The LDRI also did not differentiate left from left-lateral grafts, and included donor and recipient weight and height, instead of BSA ratios. Our models have comparable, if not better, discrimination than existing risk scores in predicting graft survival.

The use of RFS models also allowed us to consider many donor and recipient characteristics, as well as the complex interactions between them, in our algorithm. The RFS model was chosen over the Cox proportional hazard model to improve the generalizability and accuracy of the model, given the complexity of the data, and expected interactions between terms, and the potential implications these interactions can have on candidate selection for liver transplantation. The Cox proportional hazard model has difficulty with interactions between terms, making its models less interpretable and accurate (27). We included 23 different donor and recipient variables prior to variable selection to optimize the c-index. Four variables (critical ratio for D-R-BSA, malignant diagnosis, medical location of the recipient at the time of transplant, and presence of moderate ascites) were found to predict graft survival in all three types of grafts (left-lateral, left, and right). The importance of D-R-BSA ratio is not surprising as liver size matching reduces the risk of small-for-size or large-for-size syndromes, which can increase the risk of graft loss (9, 28–30). We also found that biliary atresia had selective importance in our left and left-lateral graft models, but not in the right graft model. This is explained by the majority of left and left-lateral grafts being transplanted from adult donors into pediatric recipients. Finally, unlike other risk scores, we did not include recipient age (22, 23) in our final models. Although ages of the donor and recipient were included in all 3 models (left-lateral, left, and right graft), donor age did not reach importance in any of the 3 graft models, likely due to most living liver donors being younger and healthy, with few donors being over age 50 years. Similarly, recipient age did not reach importance in either the left-lateral or left graft models. Recipient age in the right graft model had very low importance for older age, conferring a slightly poorer survival (importance of 0.0012, which was well below our threshold for assigning clinical relevance).

Our model adds to the existing literature that supports the use of artificial intelligence (AI) and machine learning models to optimize liver transplant care. Studies have already shown that AI models can improve the accuracy of predicting waitlist mortality (31) and post-transplant outcomes in deceased donor liver transplantation (DDLT). Briceno et al. described an AI model that outperformed MELD, D-MELD, and the survival outcomes following liver transplantation (SOFT) scores, in predicting 3-month liver graft survival after DDLT (32). A systematic review also found that the use of artificial neural network models better predicted graft survival after DDLT (33). In this context, our model is an important first step towards integrating machine learning techniques to improve allocation of hepatic grafts from ND-LLD. At our institution, we have successfully used this model to help guide our clinical decision-making around ND-LLD liver allocation, utilizing a 10-year graft survival threshold of 65%, based on the Adult-to-Adult Living Donor Liver Transplant Cohort Study (A2ALL) data (34).

Our study has several strengths. First, we utilized national LDLT data from the U.S. OPTN database to inform our analytic models, including adult-to-adult and adult-to-child transplants, which allows for greater generalizability of our findings. Second, in addition to developing graft survival prediction models, we also confirmed the models’ abilities to discriminate survival by comparing the accuracy of predictions to actual graft survival. Third, the use of RFS models allowed us to account for a large number of donor and recipient variables, along with their potential interactions.

We acknowledge the limitations of this study. Although our models were developed using a large, national dataset, this was a retrospective analysis. In addition, the c-index for our left and right models were <0.7, suggesting that other factors that are not currently included in the U.S. OPTN data, may improve the fit of the models (35, 36). For example, graft weight or volume for calculation of the GRWR is not available in the OPTN data, so we included BSA in our analyses since prior studies have demonstrated an increased risk of graft failure when donor and recipient BSA are mismatched. Although our c-indices for left lobe and right lobe graft survivals were in the 0.6 range, there is precedence for using modeling in transplant with c-indices in the 0.6 range, most notably in the current U.S. lung allocation system that incorporates a post lung outcome model with a c-statistic in the 0.6-0.7 range. Additionally, although 5% of LDLTs performed during the study period in the U.S. were from ND-LLDs, our models were developed using all living liver donor transplant data during the study period, regardless of whether the donor was non-directed or directed. However, since our models were developed using objective, measured factors that were demonstrated to be predictive of graft survival, it would not be expected that donors in our modeling data being ‘directed’ vs ‘non-directed’ would impact our final modeling results.

Finally, within the context of the national data used for these analyses, we could not account for individual transplant center experience, such as LDLT volume, frequency of LDLT surgery, or surgical experience, which have been shown to impact LDLT outcomes (3, 37, 38). However, our models can be individualized to each liver transplant program, such that identification of potential recipients for ND-LLD livers can be tailored to characteristics of local waiting lists, donors, and center experience.

In conclusion, there is an unmet need for more evidence to help guide living liver donor programs in the allocation of hepatic grafts from nondirected ‘anonymous’ living liver donors in the U.S. In this original, proof of concept study, our models present real world evidence from real world data to help address this unmet need, and include complex donor-recipient interactions to accurately predict 10-year graft survival for left-lateral, left, and right grafts. The model was particularly robust for left-lateral grafts. Therefore, our results demonstrate that machine learning is useful in the development and implementation of allocation models that can guide transplant clinical decision making and help optimize outcomes for recipients of ND-LLD hepatic grafts. As the opportunities for LDLT and ND-LLD donation expand, our findings set the stage for further development of robust allocation strategies for ND-LLD grafts.

## Data availability statement

Publicly available datasets were analyzed in this study. This data can be found here: https://www.srtr.org/about-the-data/the-srtr-database/.

## Ethics statement

Ethical review and approval was not required for the study on human participants in accordance with the local legislation and institutional requirements. Written informed consent from the participants’ legal guardian/next of kin was not required to participate in this study in accordance with the national legislation and the institutional requirements.

## Author contributions

KB, SB, JP, and MS contributed to the design and implementation of the research. JP, KB, and SB contributed to the analyses of the results. NK, KB, SB, JP, CK, MS, JR, PH, RB, and AD participated in critical data interpretation and writing of the manuscript. KB, SB, MS, and JP conceived the original idea and supervised the project. All authors contributed to the article and approved the submitted version.

## Glossary

**Table:**

A2ALL	Adult-to-Adult Living Donor Liver Transplant Cohort Study
AUC	Area under the curve
AI	Artificial intelligence
BAR	Balance of risk score
BSA	Body surface area
CIT	Cold ischemia time
CR	Critical Ratio for donor-to-recipient body surface area
DDLT	Deceased donor liver transplantation
D-MELD	Donor age with Model for End-Stage Liver Disease score
D-R-BSA ratio	Donor-to-recipient body surface area ratio
GRWR	Graft-to-recipient weight ratio
ICU	Intensive care unit
IQR	Interquartile range
LDLT	Living donor liver transplantation
LDRI	Living Donor Risk Index
MELD	Model for End-Stage Liver Disease score
ND-LLD	Nondirected living liver donor
OPTN	Organ Procurement and Transplantation Network
PELD	Pediatric End-Stage Liver Disease score
RFS	Random forest survival analysis
SOFT	Survival outcomes following liver transplantation
TRI	Transplant risk index
UNOS	United Network for Organ Sharing
